# A case for subnational nutrition financing: The development and use of county-level investment cases in Kenya

**DOI:** 10.1371/journal.pgph.0004128

**Published:** 2025-02-25

**Authors:** Sakshi Jain, Sameen Ahsan, Dylan David Walters, Geoffrey Kinyua, Johniere Smith, Martha Nyagaya, Alison Greig, Mandana Arabi

**Affiliations:** 1 Nutrition International, Ottawa, Ontario, Canada; 2 Nutrition International Kenya, Nairobi, Kenya; Dalhousie University, CANADA

## Abstract

This paper aims to emphasize the significance of creating subnational nutrition action plans in regions with high variation in nutrition challenges and evaluates their projected return on investment in Kenya. Despite steady progress, undernutrition in Kenya remains high, costing the country an estimated US$ 4.2 billion or 7% of its GDP annually. Under Kenya’s decentralized government system, numerous counties developed sectoral County Nutrition Action Plans (CNAPs) in 2018 to identify and prioritize essential nutrition actions to target undernutrition at the subnational level. In this paper, the authors present findings from county investment cases (CICs) in five counties — Nandi, Busia, Makueni, Vihiga, and Elgeyo Marakwet—including the costs, health impacts, and benefit to cost ratios of implementing high-impact nutrition interventions. Data was collected on the target coverage and cost of interventions prioritized in each county’s CNAPs for the 2018 to 2022 period. A monetized DALY approach, using the value of a statistical life methodology was used for cost-benefit analysis and the Optima Nutrition tool was used for cost-effectiveness analysis. The estimated cumulative impact of the five CNAPs was projected as 1,800 child and 115 maternal deaths averted; preventing and treating 19,000 cases of stunting and 4,700 cases of wasting in children under five and averting 67,000 cases of anaemia in pregnant women and adolescent girls. The county-level benefit-cost ratios range from $5:1 to $14:1 (at a default 3% discount rate). This analysis demonstrates that localized subnational plans can be advantageous for policymaking and prioritization to better address subnational disparities in undernutrition and offer a high return on investment.

## Introduction

In the last two decades, Kenya has shown steady progress in reducing undernutrition. Between 1998 and 2014, the prevalence of stunting among children under five years decreased from 33% to 26%, wasting reduced from 6% to 4%, and underweight decreased from 22% to 11% [1]. Presently, Kenya is on course to meet four global nutrition targets on under-five stunting, wasting, overweight, and exclusive breastfeeding [2].

Globally, undernutrition costs the world at least US$ 761 billion per year, or US$ 2 billion per day, equivalent to 1% of global income [3]. While progress has been significant, undernutrition remains unacceptably high in Kenya. A 2019 Cost of Hunger in Africa study estimated that in 2014, the effect of undernutrition on health, education, and productivity amounted to about US$ 4.2 billion or roughly 7% of Kenya’s 2014 GDP [4].

In 2014, 26% of Kenya’s 7.22 million under-five population (approximately 1.8 million children) were stunted [1], and county-level stunting prevalence ranged from 15% in Nyeri to 46% in West Pokot [5]. High stunting levels were found in the Coast, Eastern, and Rift Valley regions, whereas the highest wasting prevalence was observed in the Northern Region [5]. Of Kenya’s 47 counties, 19 had a stunting prevalence above 30%, and wasting prevalence ranged from 0.2% in Siaya to 23% in Turkana [5]. High regional variation results from differences in poverty levels, food insecurity, education, access to water and sanitation, access to and use of health services and infrastructure, and governments’ budget restraints across regions. It is important to note that these estimates are from before the onset of the COVID-19 pandemic, which caused widespread unemployment, food insecurity, and disruptions to health systems [6], with facility closures [7] and treatment unavailability [8] contributing to an estimated additional 450–2,120 child deaths and 38–196 maternal deaths monthly in Kenya [9].

Unfortunately, the trends in gross official development assistance (ODA) and nutrition-financing have not followed this increasing need. Even at its highest, nutrition-related ODA was less than 1% of the total ODA in Kenya, despite high returns on investment [10]. A 2016 analysis by the World Bank estimated that if critical high-impact nutrition interventions are scaled up in Kenya, the benefit-cost ratio of the investment would be 22:1, i.e., every dollar invested in nutrition interventions would potentially result in $22 in economic returns [11].

In Kenya, public health care delivery is devolved; the 2010 Constitution gives Kenya’s 47 counties responsibility for delivering most health services while the national government retains leadership in developing health policy and regulation and in managing national referral facilities. Given the decentralized system of governance in the country, the significant subnational variation observed in trends of undernutrition, and the limited ODA allocated to Kenya, it has increasingly become essential that county governments spearhead financing nutrition interventions.

As a part of their response to this need, in 2018, the Government of Kenya developed its second National Nutrition Action Plan. The Kenya Nutrition Action Plan 2018-2022 (KNAP) utilized existing country-level policy and regional frameworks to outline the investment required to address malnutrition in all its forms and for all ages. The KNAP provided an umbrella framework and guidance for developing County Nutrition Action Plans (CNAPs) [1]. The costed CNAPs were created to tailor essential nutrition actions to meet the specific needs of individual counties and prioritize accordingly - as well as leverage additional county-level resources for nutrition. The high-impact nutrition interventions included in the CNAPs were the same as those identified in the 2016 World Bank report [11].

The CNAPs were created by the country’s two levels of government in collaboration with key line ministries, development partners, NGOs, and civil society organizations. The authors’ organization and other development partners supported county governments with technical and financial support for the CNAPs. In addition, County Investment Cases (CICs) were created to estimate the potential health impacts and economic benefits of investing in low-cost, high-impact direct interventions included in the CNAPs. The primary goal of the development of the CICs was to mobilize county governments to increase investment in nutrition actions by illustrating the connection to human well-being and economic development.

This complemented Kenya’s Universal Health Coverage (UHC) policy [12] which strives to ensure that all Kenyans can access quality health services without financial hardship, guided by the principle of “leaving no one behind.” Achieving this vision requires addressing key health determinants, with nutrition recognized as a foundational and cost-effective intervention to improve health outcomes across all populations. The UHC Policy 2020–2030 highlights the importance of multisectoral approaches and prioritizing preventive and promotive health strategies. In this context, investing in low-cost, high-impact nutrition programs at the county level offers a strategic opportunity to advance UHC goals.

Investment cases for development sectors, including health and nutrition, have been used increasingly globally and nationally in the last decade. Yet, few examples exist of the approach being applied to subnational decision-making. A secondary goal for this exercise was to develop a consistent data driven methodology to project health impacts and conduct benefit-cost analysis on investment of subnational nutrition plans. While many studies and analyses have tried to estimate the returns on investment in nutrition [13,14], each study applies a different methodology and uses data of varying degrees of quality, resulting in different conclusions. Applying a consistent methodology to estimate the economic returns on allocating resources for the implementation of CNAPs allows for better comparison across geographies.

This paper aims to present both the quantitative results of the CICs for five counties that launched their CNAPs (Busia, Elgeyo-Marakwet, Makueni, Nandi, and Vihiga) and discuss the lessons learned from the process to add to the knowledge base on the value of and the methods for economic evaluation of nutrition interventions at the subnational level.

## Methods

The CICs included two key steps - projecting the forward-looking health impacts of the proposed multi-year CNAPs, and conducting benefit-cost analysis and cost-effectiveness analysis. Ideally, investment cases for nutrition could also include a form of optimization analysis, which factors in budget constraints and/or priority outcomes to generate a recommended optimal set of interventions for investment and a few proposed realistic financing scenarios for policymakers and investors to consider. These two steps were not included in Kenya’s CNAP and CIC process.

The five counties—Nandi, Busia, Makueni, Vihiga, and Elgeyo Marakwet—were selected because they were the pioneers in spearheading the creation of County Nutrition Action Plans (CNAPs). These counties were the first to be ready to initiate the CNAP development process, positioning them ahead of other counties in the implementation timeline. County governments undertook a detailed exercise to develop fully costed CNAPs between 2019 and 2020. This activity was a real-world ingredients-based costing exercise designed to collect data on the costs, inputs and assumptions related to the price, quantity, and targets for every line item in the government’s budget related to nutrition. Kenya’s most recent DHS survey from 2014 (at the time of analysis in 2021) was used as the source of data for several demographic and health input parameters. The CNAPs were developed by county governments in collaboration with national governments and partner organizations. Details on the CNAPs, including methods used and assumptions made, are available in the published CNAP studies [15–19].

This CNAP costing data was reanalyzed to create the CICs to support advocacy for financing. The indicators of interest from the costing analysis were the unit cost per intervention per beneficiary, total cost per intervention, and total cost of each broad category of nutrition program.

No ethical clearance was required as this study utilized secondary, and anonymized data. The findings aim to promote equity and support underserved populations, aligning with public health objectives to improve nutrition outcomes in Kenya.

### The financing need for nutrition interventions

The CNAPs consist of many activities to address undernutrition in the counties over four years, which were categorized into the following four groups for the CIC analysis, guided by clear set of criteria (see S2 Appendix):

1. **High-impact direct intervention (HIDI) related activities**: Such interventions are aimed at addressing the immediate determinants of maternal, newborn and child nutrition and development, such as adequate food and nutrient intake, responsive feeding, caregiving and parenting practices, and low burden of infectious diseases [20]. These interventions were earlier classified as nutrition-specific interventions, and as per the latest Lancet framework, these are now called direct health sector interventions [21]. The CNAPs budgeted direct health sector interventions such as Weekly Iron and Folic Acid Supplementation in adolescents (WIFAS), Iron and Folic Acid Supplementation in pregnancy (IFAS), Infant and Young Child Feeding (IYCF) (including growth monitoring and promotion), Kangaroo Mother Care (KMC), Multiple Micronutrient Powder (MNP), Zinc and Oral rehydration solutions (ORS), Integrated Management of Acute Malnutrition (IMAM), Vitamin A Supplementation (VAS).
2. HIDI activities included training healthcare workers and community health volunteers on Integrated Management of Acute Malnutrition (IMAM), establishing mother-to-mother support groups, procuring and disseminating micronutrient capsules, conducting nutrition screenings for early identification of malnutrition, and delivering male and female healthcare worker classroom training on Kangaroo Mother Care. Other activities included biannual Vitamin A supplementation campaigns and supporting community health committees. These activities were classified as HIDI based on their direct impact on individuals receiving the interventions.
3. **Indirect nutrition-sensitive intervention related activities:** These address the underlying determinants of maternal, newborn and child nutrition and development [20]. This category includes social, and behavior change activities such as WASH and nutrition-related activities under social protection. These interventions, earlier classified as nutrition-sensitive , are now categorized as indirect health sector interventions per the latest Lancet recommendations.
4. **Enabling environment activities:** Enabling environment activities were those aimed at strengthening systems and policies to support nutrition interventions indirectly. Examples include mapping nutrition partners and stakeholders, strengthening mechanisms for policy, legal, and regulatory engagement, sensitizing private partners on county strategies for public-private partnerships, and developing resource mobilization strategies for nutrition (covering financial, human, and organizational resources). Additional activities included monitoring the implementation of the CNAP and its M&E framework and evaluating the impact of nutrition interventions within the county. These activities were categorized as enabling environment due to their focus on system-wide improvements rather than direct intervention delivery.
5. **Other public health activities:** This category included activities for senior care, clinical and community nutrition, and dietetics, advocacy for interventions not covered under the high-impact direct category, and anything related to non-communicable diseases that needed more corrective action than preventative action (such as TB, HIV, diabetes, and hypertension).

Each activity within the CNAP was very context-specific, and thus, some activities could fit within different categories. For activities that were difficult to categorize and could potentially fall into multiple categories, efforts were made to classify them under HIDI wherever possible. For example, IYCF activities and IMAM activities are often delivered together in the counties and involve overlapping components; these were categorized as accurately as possible during the classification process. This approach ensured a conservative estimate of the benefit-cost ratio by including more activities with direct measurable benefits. Given insufficient evidence and clarity on the pathways to calculate the impact of indirect interventions and general social and behaviour change interventions, the health and economic analyses in this paper focused on the high-impact direct interventions. A full list of activities for each county is available online in the published CNAP reports [15–19].

### Targets for high impact direct nutrition interventions

The baseline and target coverage (%) figures from the CNAPs were used to estimate the cumulative persons reached by each CNAP intervention. The baseline and target coverage rates from the CNAPs for each HIDI are shown in Table 1, and we assumed a linear scaling of coverage between those years. The calculation of the unit costs of each intervention was based on the sum of the cost of the ingredients divided by the total number of target beneficiaries over the CNAP’s time frame. With 2018 as the baseline year as per the CNAPs, the costing and subsequent impact analyses were conducted for a 4-year timeframe from 2019 to 2022.

**Table 1:** CNAP targets for high-impact nutrition interventions (%).

Intervention	Target Beneficiary	Nandi		Vihiga		Busia		Makueni		Elgeyo Marakwet	
		2018	2022	2018	2022	2018	2022	2018	2022	2018	2022
**WIFAS**	Adolescent girls in school (10-19 yrs) supplemented with WIFAS	0%	75%	0%	60%	90%	95%	0%	11%	0%	25%
**IFAS**	Pregnant women attending at least 1 antenatal counselling session	87%	90%	85%	95%	81%	90%	84%	90%	56%	80%
**KMC**	Low-birth weight babies that received KMC	NA*	NA*	2%	4%	NA*	NA*	NA*	NA*	1%	8%
**IYCF** ^†^	Children under 23 months whose caregivers received breastfeeding promotion	0%	96%	0%	96%	0%	100%	0%	98%	100%	100%
**MNPs** ^‡^	Children 6-23 months receiving MNPs	NA*	NA*	0%	25%	NA*	NA*	0%	25%	27%	60%
**VAS**	Children 6-59 months receiving 2 dose Vitamin A Capsule supplementation	68%	80%	26%	80%	100%	100%	100%	100%	33%	70%
Zinc + ORS	Children under 5 years who suffer from diarrhoea receiving zinc + ORS treatment	NA*	NA*	NA*	NA*	NA*	NA*	100%	100%	89%	95%
**Treatment of IMAM** ^‡^	Children U5 suffering from IMAM receiving treatment	0%	80%	0%	80%	0%	80%	0%	80%	0%	80%

Abbreviations: WIFAS, weekly iron and folic acid supplementation in adolescents; IFAS, iron and folic acid supplementation in pregnancy; KMC, kangaroo mother care; NA, not applicable, IYCF, infant and young child feeding; MNPs, multiple micronutrient powder; VAS, vitamin A supplementation; ORS, oral and rehydration solutions; IMAM, integrated management of acute malnutrition.

*Not all interventions were proposed in the CNAP for each county. For such interventions, the target coverage for the respective counties have been left blank (for example, there was no proposed MNP plan in Nandi).

Where no baseline coverage data existed, the authors assumed 0% coverage at baseline.

^†^IYCF – the CNAPs included targets for the prevalence of exclusive breastfeeding, so coverage targets were calculated using estimates from Sinha et al.’s 2015 [22] paper.

^‡^No coverage indicators were included in CNAPs for MNPs and Treatment of IMAM; common coverage targets were set for all counties that had budgeted for MNP intervention and Treatment of IMAM in consultation with CNAP consultants in Kenya.

Source: CNAPs and consultations with CNAP consultants in Kenya.

### Health impact analysis

The health impacts of implementing high impact direct interventions as per the CNAP targets in each county were estimated using the Optima Nutrition modelling tool [23]. The primary health outcomes of interest in this analysis were cases of stunting averted in children (6-59 months), cases of wasting averted in children (6-59 months), child deaths averted, maternal deaths averted, and cases of anaemia averted in adolescent girls (15-19 years), and pregnant women (15-49 years).

While some interventions such as IFAS, VAS, and IYCF were already established in the counties prior to 2018, with varying levels of coverage, this analysis compared the total cost (budget impact) and total health outcomes estimated at the CNAP target coverage level in each year to a scenario of no coverage without CNAPs, rather than only the incremental costs and health outcomes. This approach allowed the authors to present a holistic assessment of the potential benefits associated with the CNAPs to inform evidence-based decision making and resource allocation from the county governments. The health impact generated was calculated as:

#### Projected health outcomes (cases averted): Zero coverage – CNAP Target coverage.

The projected health impacts were then converted into disability-adjusted life years (DALYs). DALYs averted were calculated as the sum of years lived with disability (YLDs) averted, which refers to total morbidity averted due to stunting, wasting, and anaemia, and Years of life lost (YLLs) averted, which refers to the total number of years of expected life lost due to premature mortality. To estimate YLLs averted, the product of the number of cases of undernutrition-related preventable child deaths and the expected lifespan of the child were calculated while accounting for age at averted death. The YLD averted were calculated by taking the product of the number of cases of a nutritional deficiency, the corresponding disability weight of the deficiency (0.02 for stunting, 0.051 for wasting and 0.052 for anaemia), and the duration of the effect of the intervention [24,25]. Stunting and wasting intervention effects were expected to last a lifetime, while anaemia interventions were expected to be effective for one year.

It must be noted here that it is likely that there is a proportion of the under 5 population that suffers from multiple nutritional deficiencies. It was not possible to estimate the number of children covered that had multiple anthropometric deficits, but evidence suggests that children with multiple anthropometric deficits are likely to have more severe cases of stunting and wasting than those cases with stunting or wasting only, and likely should be assigned a higher disability weight [26]. Thus, to keep estimates conservative, moderate disability weight estimates were used for both stunting and wasting cases averted.

### Cost-effectiveness and benefit-cost analyses

Cost-effectiveness analysis provides a method for comparing the value of an intervention in terms of saving lives or improving lives, which can help identify interventions that can yield the greatest improvement in health for the least resources. The cost per DALY averted by county indicator was calculated separately by dividing the total number of DALYs averted by the total program cost.

Benefit-cost analyses provide a method for estimating the long-term economic return on investing in an intervention. This analysis utilized a monetized DALY approach using the value of a statistical life [27,28]. This approach estimated the approximate economic value of the burden of morbidity and mortality averted due to interventions proposed in the CNAPs as the product of the number of DALYs averted [29] and the value of a statistical life for Kenya (US $ 231,000 from 2015 adjusted to 2018) [28]. DALYs averted were calculated in terms of United States dollars (USD) at the county and aggregate levels for 2017. The exchange rate (KSh to USD) used throughout the analysis was 1 KSh = 0.0096 USD.

The benefit-cost analysis adhered to the methods outlined in the *Reference Case Guidelines for Benefit-Cost Analysis in Global Health and Development* (2019) by Robinson et al. [29], supported by the Bill & Melinda Gates Foundation. For the analysis, it was assumed that the effects of anaemia prevention interventions last for one year and thus only avert cases of anaemia for one year. Averting stunting and wasting leads to survival and health benefits that extend far into the future of a child’s life, and thus, it is important to discount the monetary value of health benefits to translate them into present value. For the primary analysis in this paper, a commonly used 3% default discount rate on the number of DALYs averted was used, and alternative 0% and 5% discount rates were calculated for the sensitivity analysis (see S1 Table). For the final benefit-cost ratio calculation, benefits included the total monetized benefits from DALYs, and costs included the total cost of the high-impact intervention package for each CNAP, including other high-impact direct costs.

## Results

The five counties were chosen to create investment cases due to their high burden on undernutrition. Evidence shows that those who suffer from undernutrition and diseases are more likely to repeat grades in school and experience lower productivity levels into adulthood [30]. Table 2 presents the prevalence of undernutrition in the five focus counties in 2014 showing that all counties experienced high rates of stunting and anaemia in children. While county level data was not available for prevalence of anaemia in women, regional estimates suggest that the counties experienced between 40-55% anaemia rates among women.

**Table 2:** Annual cases and costs of undernutrition by county.

	Nandi	Vihiga	Busia	Makueni	Elgeyo-Marakwet
**Prevalence of low birthweight**	6.6%	4.8%	4.8%	8.4%	6.6%
**Underweight prevalence**	11.1%	5.9%	9.0%	10.2%	12.6%
**Stunting prevalence**	29.9%	23.5%	22%	25.1%	29.9%
**Percentage of children with acute respiratory infections**	7.0%	16.6%	6.2%	10.3%	9.4%
**Percentage of children with fever**	19.7%	49.2%	42.7%	14.1%	29.7%
**Percentage of children diarrhea**	10.9%	23.6%	18.2%	11.3%	11.6%
**Prevalence of anaemia in children**	28.3%	36.3%	26.3%	26%	36.3%

Source: KDHS 2014 [5], Kenya Malaria Indicator Survey 2015 [31]

### The health impact of high impact nutrition interventions

Over the implementation period of the CNAPs, high-impact direct interventions are projected to prevent 1,835 child deaths across the five counties: 267 in Nandi, 276 in Vihiga, 650 in Busia, 340 in Makueni, and 302 in Elgeyo Marakwet. Additionally, it is projected that 115 maternal deaths will be prevented across the five counties: 23 in Nandi, 19 in Vihiga, 39 in Busia, 23 in Makueni, and 12 in Elgeyo Marakwet.

**Fig 1:**
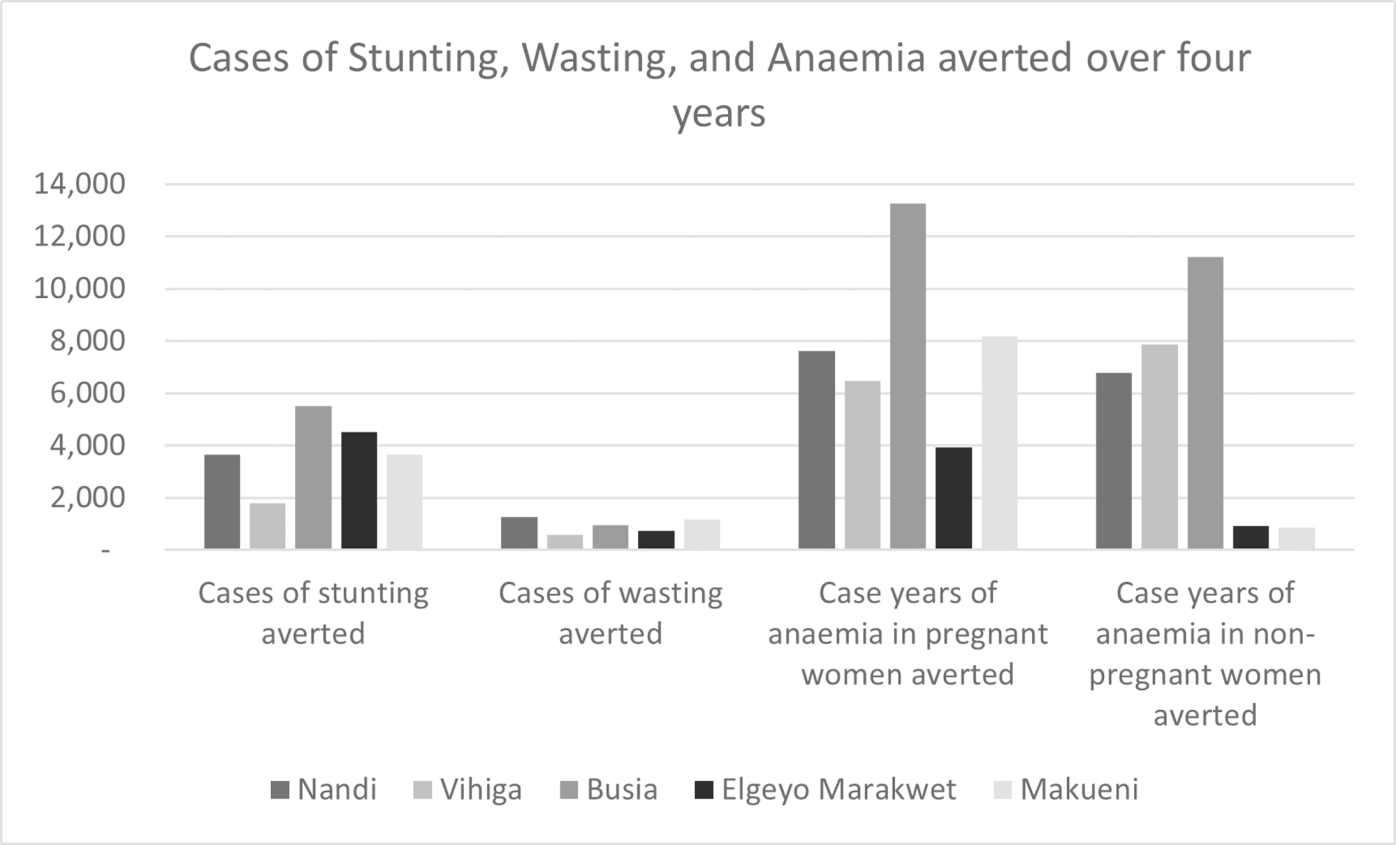
Projected cases averted due to reaching targets of interventions included in the CNAPs.

Cumulatively over four years and five counties, the projected health outcomes are estimated as approximately 19,000 cases of stunting averted, 4,700 cases of wasting averted, and 67,000 case-years of anaemia averted. In cases of anaemia, the protection is only assumed to last for one year, and ongoing supplementation would be essential to the long-term prevention of anaemia cases (Fig 1).

In total, these projected health outcomes averted by the CNAPs are equivalent to averting a total of 142,471 of DALYs across the five counties over four years, including 23,181 in Nandi, 21,151 in Vihiga 47,987 in Busia, 27,236 in Makueni, and 22,916 in Elgeyo Marakwet.

### The financing need for high impact direct nutrition interventions

**Fig 2:**
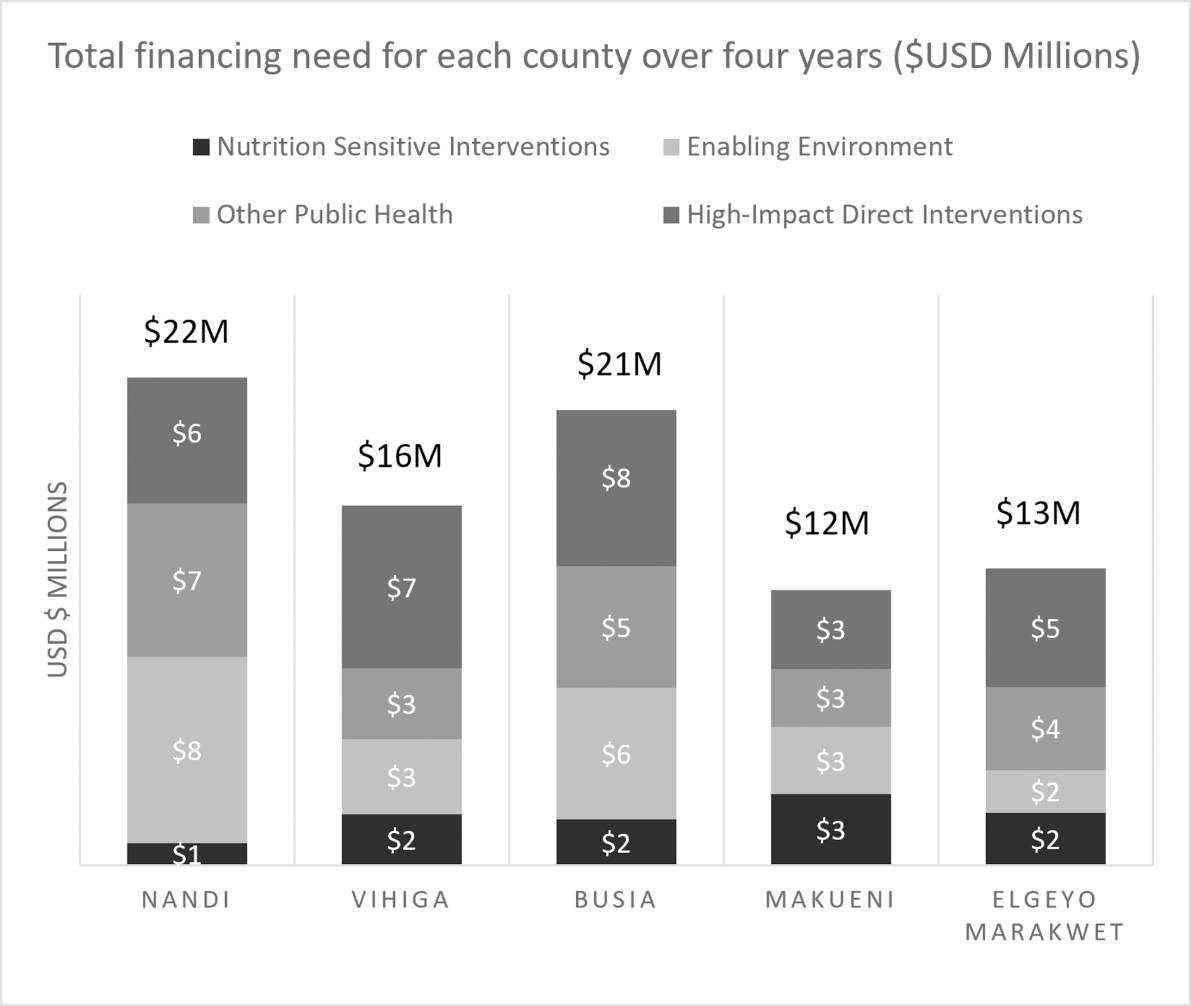
Total financing need for each county over four years by intervention type (USD $ Millions).

The total financing need for the CNAPs was approximately USD $ 17 million on average per county over four years. The costs of the interventions grouped together under the HIDI category made up the highest cost category for each county, close to 35% of the total cost (Fig 2).

Within HIDI, IYCF and IMAM programs made up most of the costs. These interventions include overlapping activities and use similar equipment, and are often carried out together in Kenyan counties. For example, children under 5 years are monitored on their height and weight and overall health as part of routine IYCF growth monitoring programs. Most cases of acute malnutrition are identified during screening in the routine growth monitoring and IYCF programs. On average, across the counties, IYCF and IMAM interventions made up about 70% of the total cost of high-impact direct interventions captured in the CNAPs. Notably, there was significant variation in the cost allocation of the other high-impact direct interventions. For example, WIFAS intervention was allotted 1% of the total high-impact direct budget for Nandi, Vihiga, and Makueni, and assigned 7% and 23% of the total cost respectively for EMC and Busia’s HIDI budget. Similarly, VAS programs made up 0.3% of the total cost for Vihiga, 6-7% for Busia, Nandi and Makueni, and about 12% of EMC's total HIDI budget. S2 Fig presents the cost breakdown of high-impact direct interventions except for IYCF and IMAM interventions to provide further detail.

Table 3 presents the unit costs of high-impact direct interventions by county, compared with national-level unit costs [11] and global unit costs [32]. Generally, the unit costs align with global estimates from the literature, but there are a few significant outliers. These discrepancies can be attributed to two primary factors. First, measurement errors during the costing process may have introduced inaccuracies. Second, counties independently negotiated commodity prices with suppliers, leading to significant cost variation—some counties secured lower prices, while others faced higher costs.

**Table 3:** Calculated unit costs, cost-effectiveness, and cost-benefit values of high-impact direct interventions.

Unit cost of intervention programs (USD $ per person per year) ($ 2018)							
Intervention	Nandi	Vihiga	Busia	Makueni	Elgeyo Marakwet	Kenya	Global
**WIFAS**	$0.35	$0.41	$4.02	$0.77	$12.94	Not available	$1.45
**IFAS**	$13.60	$6.92	$2.18	$2.19	$4.68	$2.26	$13.78
**KMC**	NA	$120.08	NA	NA	$34.02	Not available	Not available
**IYCF**	$33.06	$119.74	$24.60	$9.69	$28.24	$6.90	$8.12*
**MNPs**	NA	$97.89	NA	$18.53	$6.85	$2.00	$23.71^†^
**VAS**	$1.01	$0.11	$1.08	$0.44	$4.45	$0.44	$1.36
**Zinc** ^+^**ORS**	NA	NA	NA	$0.08	$1.72	$1.34	$2.06
**IMAM**	$282.57	$357.85	$281.90	$309.90	$279.28	$48-83.32^‡^	$246.99^§^
**Cost-effectiveness and Cost-Benefit ratios for all interventions**							
	**Nandi**	**Vihiga**	**Busia**	**Makueni**	**Elgeyo Marakwet**		
Gross County Product per capita ($ 2019)	$1,249	$1,034	$731	$891	$1,733		
DALY averted	23,181	21,151	47,987	27,236	22,916		
Cost per DALY averted ($ 2019)	$269	$337	$162	$127	$226		
Benefit-cost ratio (3% discount rate^§^) ($ 2019)	7:1	5:1	11:1	14:1	8:1		

Abbreviations: WIFAS, weekly iron and folic acid supplementation in adolescents; IFAS, iron and folic acid supplementation in pregnancy; KMC, kangaroo mother care; NA, not applicable; IYCF, infant and young child feeding; MNPs, multiple micronutrient powder; VAS, vitamin A supplementation; ORS, oral and rehydration solutions; IMAM, integrated management of acute malnutrition.

*Infant and young child feeding education;

^†^Lipid-based nutrition supplements;

^‡^Outpatient programs only;

^§^IMAM interventions as proxies.

Source: Author calculations; Global values are from An Investment Framework for Nutrition, World Bank Group (2017) [14];

^††^The Global Framework presents values for Antenatal Micronutrient Supplementation for pregnant women, using it as a proxy comparator for IFAS (pregnant women) costs. Details on the sensitivity analysis of benefits and costs are presented in S1 Table

Additionally, differences in the proposed reach of interventions played a critical role in unit cost variation. For counties like Nandi and Vihiga, where the proposed reach increased dramatically (e.g., from 0% to 75% coverage), unit costs were lower due to economies of scale. Conversely, in counties where the proposed reach was relatively small (e.g., from 90% to 95% coverage or 0% to 25% coverage), the total reach was smaller, but costs remained similar to those of other counties, resulting in higher unit costs.

The lack of standardized commodity costs, combined with discrepancies in program scale and proposed reach, inherently shaped the data. These subnational unit cost calculations, therefore, provide a nuanced and context-specific understanding of intervention costs while highlighting the challenges of working with real-world, diverse datasets.

### The cost-effectiveness and benefit to cost ratios of high impact direct nutrition interventions

The calculated cost per DALY averted value for the HIDI package proposed in the CNAPs ranges from 0.13 to 0.32 times the Gross County Product (GCP) per capita for five counties. The biggest driver behind the cost per DALY averted is the cost per unit of the IMAM and IYCF interventions as they are allocated the greatest portion of the budgets. The cost per DALY averted for the high impact direct nutrition interventions indicates that the interventions were highly cost effective per the WHO CHOICE methodology, which suggests that an intervention is considered to be very cost-effective if the cost per DALY averted is less than the GDP per capita [11]. Another evaluation approach suggests that for countries with medium HDI values, cost-effective interventions are ones where the cost per DALY averted is less than 0.67 times the GDP per capita [33]. Thus, by both accounts this analysis finds that these interventions are, in general, expected to be highly cost-effective interventions.

For the benefit-cost analysis for each of the five counties, the benefit-to-cost ratio was positive and relatively high. In the default scenario with 3% discount rate, the benefit-to-cost ratio for the CNAP interventions ranged from $14:1 for Makueni to $5:1 for Vihiga (Table 3). Thus, for every dollar spent on high-impact direct interventions by the county government, they are projected to generate a minimum of $5 in economic benefits in the long-run.

In the sensitivity analysis, benefit-cost ratios were also calculated at a 0% and 5% discount rate. Even with discounting at 5%, each dollar invested in high-impact direct interventions is projected to generate a benefit of $4 at minimum.

It is crucial to note that the cost-effectiveness and benefit-cost analyses were only conducted for high-impact direct interventions included in the CNAPs, not the full cost of implementing the CNAP including the nutrition-sensitive, enabling factors, and other public health interventions. The combined results of the cost-effectiveness and benefit-cost analyses suggest that investing in these high-impact direct-interventions (HIDI) is good value for money at the county-level in Kenya.

## Discussion

The results of the County Investment Case (CIC) analyses in Kenya, across five counties, describe a consistent pattern showing that high-impact direct nutrition interventions will drastically save and improve lives in a cost-effective manner. The actual health impact of the CNAP investment could be possibly be much higher if the nutrition sensitive, other public health, and enabling environment investments (i.e., infrastructural costs, such as buying equipment, building breastfeeding rooms in facilities) were included in the analysis. Furthermore, the investment is expected to generate savings in the health and education sector and increase productivity in the long run. The default discount scenario (3%), with many conservative assumptions included, suggests that the CNAPs will generate a high projected benefit-cost ratio that ranges between $5-14 for every dollar invested. This aligns with broader evidence from similar studies, which found benefit-cost ratios in the range of 5.2 - 19.4 in different contexts, respectively, for similar nutrition interventions [34,35] and, for Kenya, a benefit-cost ratio of 5.8 for priority nutrition packages as estimated in the WBG Investment Framework 2024 [36]. Our analysis adds to the literature by focusing on subnational planning that considers differences between counties, supports decision-making in Kenya’s decentralized system, and uses a consistent method to compare the economic benefits of CNAP investments across regions.

The CNAP process in Kenya is the first time such detailed costing investment data have been made available at the county level in Kenya. While there is much that can be improved in the CNAP process, this first iteration was important for policymakers’ engagement in the nutrition planning and financing process. This analysis also demonstrated that there is a significant amount of variation at the subnational level in terms of the burden of undernutrition, the priorities selected by subnational policymakers and stakeholders, and the unit cost of nutrition interventions. As a result, there is variation in the cost-effectiveness across counties, which suggests that each county requires a unique optimal mix of interventions to include in their financing strategy.

The authors must note that while the analysis presented in this paper was conducted in 2019 with data from Kenya Demographic Health Survey (KDHS) 2014, newer data is now available through the KDHS 2022.The newer data shows that the burden of stunting has significantly decreased in the 5 focus counties, though prevalence of wasting and low birthweight still remain high.

Future CNAP processes in Kenya, and around the world, could be improved in several ways if the following limitations are addressed: First, the authors do not account for externalities such as inter-county migration rates, any disruptions to the health system (such as a pandemic), and effects of climate change on nutrition, while calculating the health impacts of the activities included in the CNAPs. The focus counties have some of the highest rates of net negative migration in the country (the highest being 10%) [37]. However, even accounting for this net negative migration across the counties, the interventions maintain high benefit-to-cost ratios, ranging from $4-12 return per dollar invested. Investing in public nutrition would still yield a positive return. Second, the strength of county-level analyses depends on the availability and quality of data at the county level. Certain analyses did not have data available for key inputs into the models at the county level, and therefore used proxies from national level. Using county level data will generate a better overall picture of the state of undernutrition in the counties. Third, the authors assumed each case of stunting and wasting averted as unique, i.e., the analysis did not account for the possibility that the same child may be likely to be stunted or wasted more than once during the CNAP timeframe. Fourth, the analysis showed high variation in unit costs across the counties. While a common costing framework was applied in the project, in-depth exploration of the costing data shows that in many cases, the methods for costing were not consistently applied across counties, and input costs varied drastically across counties. Integrating a review and feedback process may decrease some of the variation in costs and improve the quality of CNAPs altogether. Fifth, the CNAP process highlighted variation in costs for commodities, which justified several counties coordinating themselves in 2021 to do pooled procurement of commodities at an optimal cost via the Kenya Medical Supplies Authority. Further comparative analysis of the costing data across counties could lead to other important lessons learned and cost-saving approaches. Sixth, the CNAPs included activities for a wide-spectrum of nutrition actions, not only evidence-based high-impact low-cost nutrition interventions. Future CNAP processes could include either a prioritization process to identify the most cost-effective nutrition actions to invest in before the costing exercise, or an optimization analysis at a later stage that generates recommendations for an investment strategy given budget constraints or specific goals from policymakers, and readjust aspirational targets for scaling set earlier to more realistic levels.

In addition, the CNAPs filled an essential gap in the nutrition planning process for counties, however it led to a plan that was too aspirational. For example, the total costs of the CNAPs presented in this analysis make up about 20-22% of the total fiscal budget of many counties. Within this, the estimated costs of the activities amount to, on average, 60% of the counties’ health budgets [38–42], with significant variation observed across counties. In Kenya, counties aim to allocate 15% of their total budgets to the health sector, and at present, most counties are not able to meet this criterion. Particularly in increasingly constrained fiscal environments, future CNAPs must focus on allocating investments to high-impact nutrition interventions that are of the most value to the county and should include another step of review to ensure that the CNAP targets are plausible and that the subsequent financing need is realistically affordable for county policymakers.

## Conclusion: application of CICs in future

The CICs are a powerful tool that can be used for resource mobilization for nutrition at the county level. Between 2020 and 2022, the authors’ organization first used the CICs to build a case on the consequences of not investing and the projected returns on high impact nutrition interventions in discussions with decision-makers at county level to lobby for increased allocation of country budgets to nutrition. As a result of these deliberations, county governments ratified a three-year joint financing agreement with the authors’ organization for implementation of low-cost high impact nutrition interventions at scale.

There is high potential for applying CICs for knowledge sharing, advocacy, and policymaking to inform and influence investments in nutrition. It is often difficult to present the scale of the loss to a region’s productivity due to undernutrition and to quantify the potential impact to be gained from investing in nutrition. The CICs fill this crucial gap. Additionally, given that most existing analyses often focus on national-level results, the CICs highlight how the need for resources and potential health impacts vary considerably across different contexts. It may be recommended to also to integrate subnational county nutrition action planning (CNAPs and CICs) into the national nutrition planning process (i.e., KNAP 2023-2027) to promote bidirectional cohesiveness in the strategy, financing and implementation as seen in some other countries such as the Philippines [43]. Integration of nutrition planning processes at various government levels with a standard approach along with incorporating improved costing, prioritization and optimization feedback loops would be recommended to increase the allocative efficiency of nutrition investments in the future. In recent years, there has been an increasing focus on generating data for decision-making and financing, so this analysis for Kenya provides a framework and case study for undertaking future CICs as part of a domestic resource mobilization process in alignment with the current global focus.

## Supporting information

S1 Appendix Components of a nutrition investment case. (DOCX)

S2 Appendix Decision tree for the cost- categorization of nutrition-related activities in CNAPs. (DOCX)

S1 Fig Cumulative reach for high-impact direct programs 2018-2022. (TIFF)

S2 Fig Financing need of key HIDI interventions as a percentage of total HIDI financing need. (TIFF)

S1 Table Cost benefit – sensitivity analysis. (DOCX)
