# Supplementary figures and images for: A case for subnational nutrition financing: The development and use of county-level investment cases in Kenya
Source: PLOS Glob Public Health. 2025 Feb 25;5(2):e0004128. doi: 10.1371/journal.pgph.0004128 (PMC11856574; doi:10.1371/journal.pgph.0004128)

**S1 Appendix. Components of a Nutrition Investment Case**


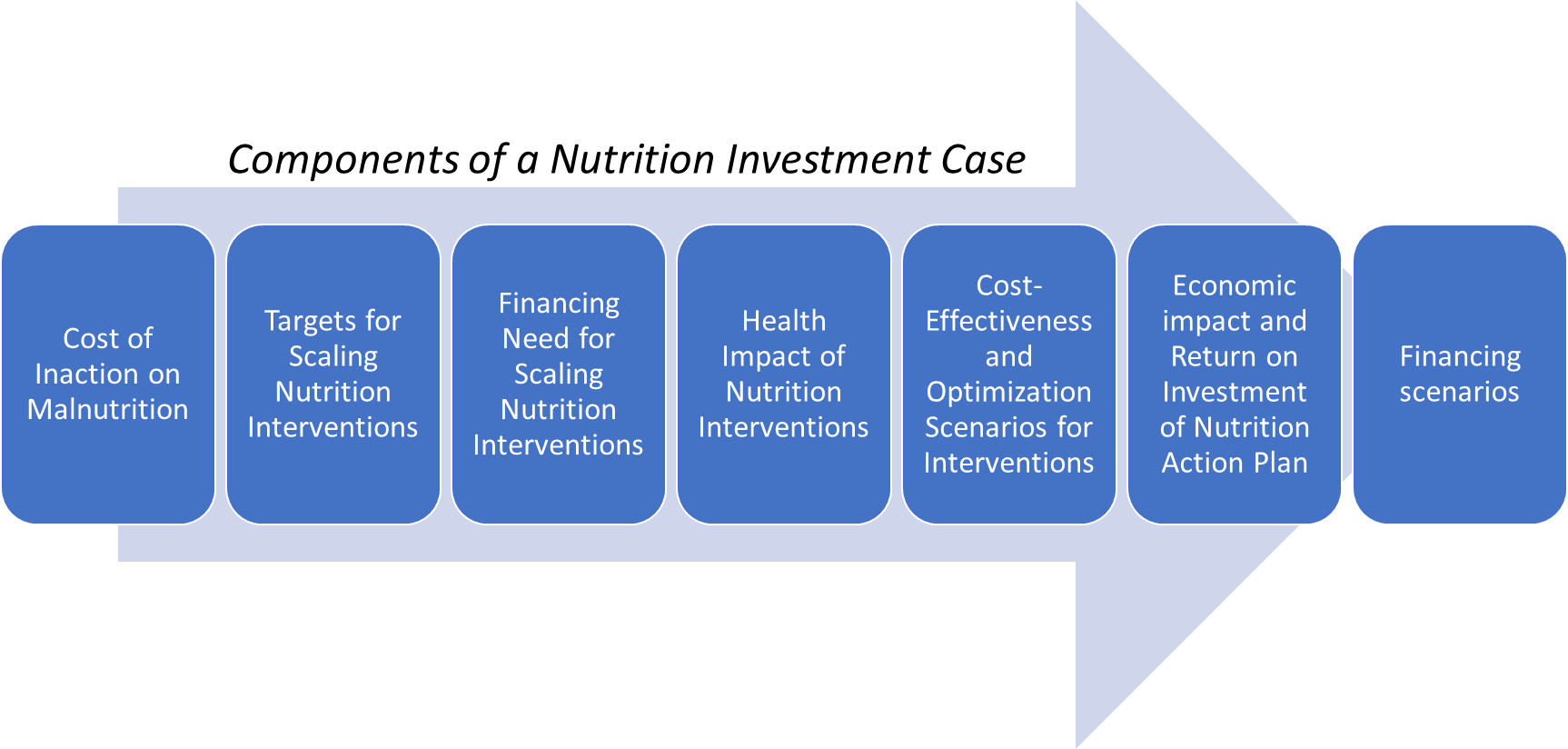

Supplement: S1 Appendix — (DOCX) [file pgph.0004128.s001.docx]

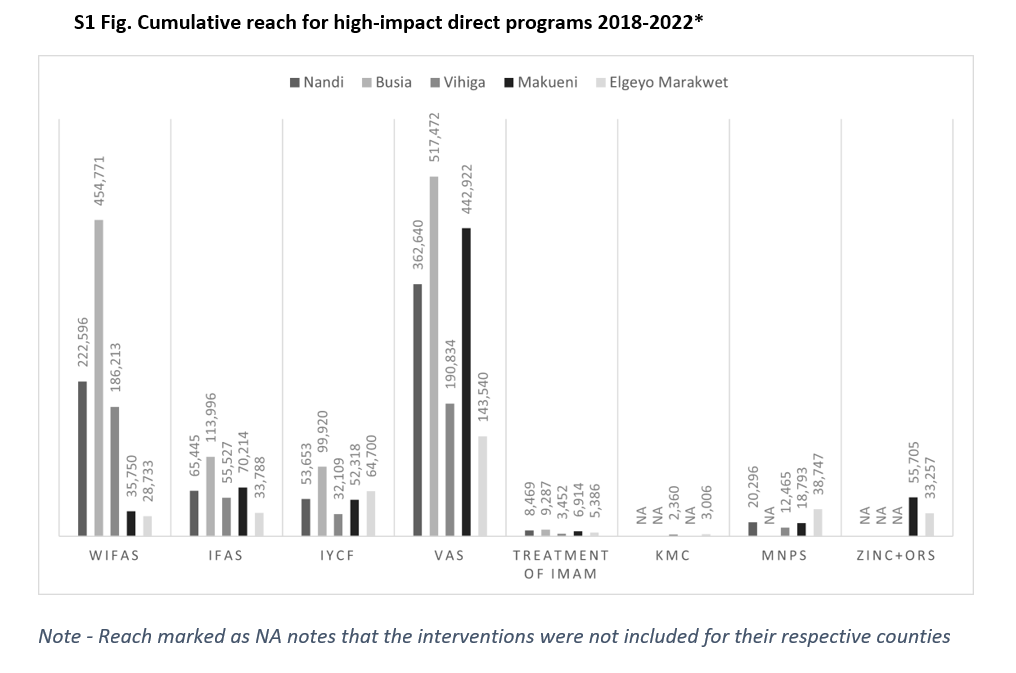

Supplement: S1 Fig — (TIFF) [file pgph.0004128.s003.tiff]
