# Supplementary material for: A case for subnational nutrition financing: The development and use of county-level investment cases in Kenya
Source: PLOS Glob Public Health. 2025 Feb 25;5(2):e0004128. doi: 10.1371/journal.pgph.0004128 (PMC11856574; doi:10.1371/journal.pgph.0004128)
Supplement: S2 Appendix — (DOCX) [file pgph.0004128.s002.docx]

**S2 Appendix. Decision tree for the cost- categorization of nutrition-related activities in CNAPs.**
