# Supplementary material for: A case for subnational nutrition financing: The development and use of county-level investment cases in Kenya
Source: PLOS Glob Public Health. 2025 Feb 25;5(2):e0004128. doi: 10.1371/journal.pgph.0004128 (PMC11856574; doi:10.1371/journal.pgph.0004128)
Supplement: S1 Table — (DOCX) [file pgph.0004128.s005.docx]

**S1 Table. Cost Benefit – Sensitivity Analysis**

| **DALYs, Monetized DALYs, Benefit-Cost Ratio and Cost Per Case Averted (USD $)** | | | | | |
| --- | --- | --- | --- | --- | --- |
|  | **Nandi** | **Vihiga** | **Busia** | **Makueni** | **Elgeyo-Marakwet** |
| DALYs averted over four years | 23,181 | 21,151 | 47,987 | 27,236 | 22,916 |
| Monetized DALYs averted (0%) | $85.72M | $78.22M | $177.46M | $100.72M | $84.74M |
| Monetized DALYs averted (3%) | $37.92M | $34.63M | $78.42M | $44.21M | $36.86M |
| Monetized DALYs averted (5%) | $25.70M | $23.48M | $53.07M | $29.73M | $24.64M |
| Cost of CNAP (0%) | $6.24M | $7.12M | $7.80M | $3.45M | $5.18M |
| Cost of CNAP (3%) | $5.87M | $6.67M | $7.35M | $3.23M | $4.89M |
| Cost of CNAP (5%) | $5.65M | $6.39M | $7.08M | $3.09M | $4.72M |
| Benefit-Cost Ratio (0%) | 14:1 | 11:1 | 23:1 | 29:1 | 16:1 |
| Benefit-Cost Ratio (3%) (Default discount) | 7:1 | 5:1 | 11:1 | 14:1 | 8:1 |
| Benefit-Cost Ratio (5%) | 5:1 | 4:1 | 7:1 | 10:1 | 5:1 |
